# Crystal Structure of Chloroplastic Thioredoxin f2 from *Chlamydomonas reinhardtii* Reveals Distinct Surface Properties

**DOI:** 10.3390/antiox7120171

**Published:** 2018-11-23

**Authors:** Stéphane D. Lemaire, Daniele Tedesco, Pierre Crozet, Laure Michelet, Simona Fermani, Mirko Zaffagnini, Julien Henri

**Affiliations:** 1 Laboratoire de Biologie Moléculaire et Cellulaire des Eucaryotes, Institut de Biologie Physico-Chimique, Unité Mixte de Recherche 8226 CNRS Sorbonne Université, 13 rue Pierre et Marie Curie, 75005 Paris, France; lemaire@ibpc.fr (S.D.L.); crozet@ibpc.fr (P.C.); michelet@ibpc.fr (L.M.); 2 Bio-Pharmaceutical Analysis Section (Bio-PhASe), Department of Pharmacy and Biotechnology, University of Bologna, via Belmeloro 6, 40126 Bologna, Italy; daniele.tedesco@unibo.it; 3 Department of Chemistry “Giacomo Ciamician”, University of Bologna, via Selmi 2, 40126 Bologna, Italy; simona.fermani@unibo.it; 4 Laboratory of Molecular Plant Physiology, Department of Pharmacy and Biotechnology, University of Bologna, via Irnerio 42, 40126 Bologna, Italy

**Keywords:** thioredoxin, Calvin-Benson cycle, photosynthesis, carbon fixation, chloroplast, macromolecular crystallography, protein-protein recognition, electrostatic surface, *Chlamydomonas reinhardtii*

## Abstract

Protein disulfide reduction by thioredoxins (TRXs) controls the conformation of enzyme active sites and their multimeric complex formation. TRXs are small oxidoreductases that are broadly conserved in all living organisms. In photosynthetic eukaryotes, TRXs form a large multigenic family, and they have been classified in different types: f, m, x, y, and z types are chloroplastic, while o and h types are located in mitochondria and cytosol. In the model unicellular alga *Chlamydomonas reinhardtii*, the TRX family contains seven types, with f- and h-types represented by two isozymes. Type-f TRXs interact specifically with targets in the chloroplast, controlling photosynthetic carbon fixation by the Calvin–Benson cycle. We solved the crystal structures of TRX f2 and TRX h1 from *C. reinhardtii*. The systematic comparison of their atomic features revealed a specific conserved electropositive crown around the active site of TRX f, complementary to the electronegative surface of their targets. We postulate that this surface provides specificity to each type of TRX.

## 1. Introduction

Thioredoxins (TRXs) are small oxidoreductases of 10–16 kDa exhibiting a characteristic three dimensional structure classified as TRX fold [1], composed of a single canonical globular domain comprising a mixed β-sheet surrounded by four α-helices [2,3,4]. These proteins play a key role in controlling the redox status of protein disulfide bonds in all non-parasitic organisms [5]. The redox activity of TRXs is guaranteed by the presence of a solvent-exposed motif (most commonly Trp-Cys-Gly-Pro-Cys) containing two cysteine (Cys) residues that catalyze protein disulfide reduction. TRXs are recognized as having diverse roles in numerous cellular processes and human diseases [6,7,8,9]. Non-photosynthetic organisms contain a limited number of TRXs (two in *Escherichia coli*, three in *Saccharomyces cerevisiae*, and two in *Homo sapiens*), which are localized in the cytosol and mitochondria, and are reduced by the nicotinamide adenine dinucleotide phosphate (NADPH)-dependent flavoenzyme thioredoxin reductase (NTR). By contrast, in photosynthetic organisms, TRXs are part of a large multigenic family (four in *Synechocystis* sp. PCC6803, 21 in *Arabidopsis thaliana*, and nine in *Chlamydomonas reinhardtii*). Phylogenetic and sequence analyses led to the classification of plant TRXs in different types: TRXs f, m, x, y, and z are chloroplastic, while o-type and h-type are found in mitochondria and cytosol [10,11,12,13]. Cytosolic and mitochondrial TRXs are reduced by NTRs, while chloroplastic TRXs are specifically reduced by the iron-sulfur containing ferredoxin–thioredoxin reductase, which derives electrons from ferredoxin and the photosynthetic electron transfer chain [14,15,16,17].

In photoautotrophic eukaryotes, TRXs were originally identified for their ability to modulate the activity of chloroplastic enzymes involved in carbon metabolism, such as the Calvin–Benson cycle fructose-1,6-bisphosphatase (FBPase) [18], NADP malate dehydrogenase (NADP-MDH) [19], or glucose-6-phosphate dehydrogenase [20]. In the dark, chloroplast 2-cysteine peroxiredoxins (2-CysPRX) inactivate FBPase, phosphoribulokinase (PRK), NADP-MDH, and glyceraldehyde-3-phosphate dehydrogenase (GAPDH) by oxidation [21,22]. Subsequent activation by light proceeds through the TRX-dependent reduction of regulatory disulfide bonds. Proteomic studies revealed that TRXs potentially reduce more than 1000 targets [23]. The large number of putative targets highlights the crucial role of TRXs in the control of a myriad of metabolic pathways and processes. Nevertheless, the molecular mechanisms underlying the TRX-dependent regulation of numerous targets are not clearly established.

The TRX fold is one of the most conserved throughout evolution, as suggested by paleobiochemistry [24]. Conservation of the tridimensional fold and of the active site residues account for the functional redundancy of plant TRX family members, as exemplified by the functional compensation of yeast deletion mutants by plant orthologues [25,26]. Nevertheless, several studies provided evidence for a functional specialization of TRX types for the regulation of specific targets [16]. In particular, systematic evaluation of the specificity of the different TRX types for the activation of Calvin–Benson enzymes revealed that they are all specifically or preferentially activated by f-type TRX, including 3-phosphoglycerate kinase (PGK) [27], FBPase [28,29,30], sedoheptulose-1,7-bisphosphatase (SBPase) [31,32], GAPDH [33,34,35], and PRK [33,36] (for reviews see [10,14,16]). This specificity does not appear to be linked to the redox potential of the Cys couple, which ranges from −368 mV to −336 mV at pH 7.9 for the different TRX types [29,30,37,38].

Despite the wealth of data gathered from biochemical studies on the functionality of TRXs, the molecular rules for the selective reduction of a given target disulfide by a specific TRX remain open to speculation. Understanding the physico-chemistry and structural features of TRXs hence appears as a powerful entry point to estimate the physiological significance of TRX-dependent regulation, along with specificity towards target proteins [39].

Here, we describe the novel crystal structure of chloroplastic TRX f2 (CrTRXf2), and a crystal structure of TRX h1 (CrTRXh1) from *Chlamydomonas reinhardtii*. Extensive comparison with other known 3D structures of algal or land plants TRXs and their targets allowed for structural features likely responsible for target recognition to be distinguished.

## 2. Materials and Methods

### 2.1. Cloning, Expression, and Purification of CrTRXf2 and CrTRXh1

**Figure 1:**
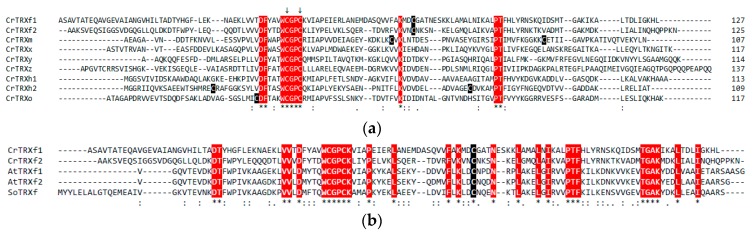
Multiple sequence alignment of plant thioredoxins (TRXs). (**a**) Sequence alignment of TRXs from *Chlamydomonas reinhardtii* (CrTRXs). CrTRXs were aligned using Clustal Omega [44,45]. The sequences used correspond to mature forms either known or predicted by the ChloroP prediction software [40]. Abbreviations and accession numbers: CrTRXf1, *Chlamydomonas reinhardtii* TRX f1 (UniProt ID: Q84XR8); CrTRXf2, *Chlamydomonas reinhardtii* TRX f2 (UniProt ID: A0A2K3DSC9); CrTRXm, *Chlamydomonas reinhardtii* TRX m (UniProt ID: P23400); CrTRXx, *Chlamydomonas reinhardtii* TRX x (UniProt ID: Q84XR9); CrTRXy, *Chlamydomonas reinhardtii* TRX y (UniProt ID: Q84XS2); CrTRXz, *Chlamydomonas reinhardtii* TRX z (UniProt ID: A8J0Q8); CrTRXh1, *Chlamydomonas reinhardtii* TRX h1 (UniProt ID: P80028); CrTRXh2, *Chlamydomonas reinhardtii* TRX h2 (UniProt ID: Q84XS1); CrTRXo, *Chlamydomonas reinhardtii* TRX o (UniProt ID: Q84XS0). An asterisk (*) indicates positions that have a single, fully conserved residue. These residues are highlighted in white on a red background. Catalytic Cys are marked with two arrows. A colon (:) indicates conservation between groups of strongly similar properties, scoring > 0.5 in the Gonnet point accepted mutation (PAM) 250 matrix. A period (.) indicates conservation between groups of weakly similar properties, scoring ≤ 0.5 in the Gonnet PAM 250 matrix. Cys residues other than the catalytic ones are highlighted in white on a black background. (**b**) Sequence alignment of *Chlamydomonas reinhardtii*, *Arabidopsis thaliana*, and *Spinacia oleracea* f-type TRXs. Mature proteins were aligned as described in panel (**a**). Abbreviations and accession numbers: CrTRXf1, *Chlamydomonas reinhardtii* TRX f1 (UniProt ID: Q84XR8); CrTRXf2, *Chlamydomonas reinhardtii* TRX f2 (UniProt ID: A0A2K3DSC9); AtTRXf1, *Arabidopsis thaliana* TRX f1 (UniProt ID: Q9XFH8); AtTRXf2, *Arabidopsis thaliana* TRX f2 (UniProt ID: Q9XFH9); SoTRXf, *Spinacia oleracea* TRX f (UniProt ID: P09856). An asterisk (*) indicates positions that have a single, fully conserved residue. These residues are highlighted in white on a red background, except the conserved non-catalytic Cys that is highlighted in white on a black background. A colon (:) indicates conservation between groups of strongly similar properties, scoring > 0.5 in the Gonnet PAM 250 matrix. A period (.) indicates conservation between groups of weakly similar properties, scoring ≤ 0.5 in the Gonnet PAM 250 matrix.

The gene at locus Cre05.g243050.t1.2 encodes chloroplastic TRX f2 from *Chlamydomonas reinhardtii* (CrTRXf2). The complementary DNA (cDNA)-encoding mature CrTRXf2 was amplified by polymerase chain reaction using 5′-AGCAAACCATGGGCGGCAGCGTTGACGGCCAG as a forward primer introducing a 5′-*Nco*I restriction site, and 5′-GGTGTGGGATCCTCAGTTCTTGGGCGGCTG as a reverse primer introducing a *BamH*I restriction site downstream of the stop codon. The cleavage site of the chloroplast transit peptide was predicted using multiple sequence alignments of plant TRX f sequences and the ChloroP prediction program [40]. Residues are numbered according to Uniprot reference sequences (ID: A0A2K3DSC9). CrTRXf2 was cloned in a modified pET-3d vector containing additional codons upstream of the *Nco*I site to express a His-tagged protein with six N-terminal histidines [41]. The expression vector was then used to transform *E. coli* BL21 Rosetta^TM^ 2 (DE3) (Novagen). Bacterial transformants were grown at 37 °C in lysogeny broth (LB) medium supplemented with 100 μg mL^−1^ ampicillin, and the production was induced at an Abs_600_ of 0.5 with 0.2 mM isopropyl-β-d-thiogalactopyranoside at 37 °C for 3 h. Cells were then harvested by centrifugation, re-suspended in 30 mM Tris-HCl (pH 7.9), and broken using a French press (6.9 × 10^7^ Pa). Cell debris were removed by centrifugation at 20,000× *g* for 20 min at 4 °C, and the supernatant was then applied onto a Ni^2+^ Hi-Trap chelating resin (HIS-Select^®^ nickel affinity gel, Sigma-Aldrich, St. Louis, MO, USA) pre-equilibrated with 30 mM Tris-HCl (pH 7.9) and 150 mM NaCl. The recombinant protein was purified according to the manufacturer’s instructions. The molecular mass and purity of the protein were analyzed by denaturing gel electrophoresis (SDS-PAGE) after dialysis against 30 mM Tris-HCl (pH 7.9) and 1 mM ethylenediaminetetraacetic acid EDTA. The concentration of purified CrTRXf2 was determined spectrophotometrically using a molar extinction coefficient at 280 nm of 17,085 M^−1^ cm^−1^ [42]. CrTRXh1 was expressed and purified, as previously described [43]. Samples of recombinant proteins were stored at −20 °C. The recombinant CrTRXf2 contains 125 residues, starting at the N-terminus with the introduced MHHHHHHHM peptide, followed by the mature protein sequences (i.e., upon removal of the chloroplast targeting sequence), beginning with a glycine (Gly65, Figure 1). Throughout the paper, residues are numbered according to the mature protein sequence (Gly65 in the preprotein becomes Gly1 in the mature protein).

### 2.2. Crystallization and Diffraction Data Collection

Sparse-matrix screening of candidate crystallization conditions was set up on iQ plates from TTP Labtech Ltd. (Melbourn, United Kingdom) with mixes of 100 nL protein and 100 nL commercial precipitant solutions (Qiagen) and incubated at 20 °C. Monocrystals of CrTRXf2 grown in condition JCSG II 26 (100 mM HEPES-NaOH, pH 7.5, 2% polyethylene glycol (PEG) 400, 2.0 M ammonium sulfate) were harvested and flash-frozen in liquid nitrogen. A complete, 2.01 Å resolution, diffraction dataset was collected on beamline ID29 at the European Synchrotron Radiation Facility (Grenoble, France). Monocrystals of CrTRXh1 grown in condition Classics 70 (200 mM ammonium sulfate, 100 mM sodium cacodylate, pH 6.5, 30% PEG 8000) were harvested and cryo-protected with an additional 25% ethylene glycol before flash-freezing in liquid nitrogen. A complete, 1.38 Å resolution, diffraction dataset was collected on beamline Proxima-1 at the SOLEIL synchrotron (Saint-Aubin Gif-sur-Yvette, France).

### 2.3. Structure Determination, Model Building, and Analysis

The native I222 dataset of CrTRXf2 crystal was used for molecular replacement by PHENIX.PHASER-MR [46], with an homology model of the protein calculated by PHYRE2 [47] and three copies of each per asymmetric unit. The top solution was refined by PHENIX.REFINE [48]; the resulting molecular model was manually adjusted into experimental electron density in COOT software [49] and further refined until reaching the final *R*-work = 0.2262 and *R*-free = 0.2638 with 98.34% favored Ramachandran dihedrals (Table 1). The native P3121 dataset of CrTRXh1 crystal was used for molecular replacement by PHENIX.PHASER-MR, with chain A from Protein Data Bank entry 1EP7.pdb [50] as a search model and two molecules per asymmetric unit. The top solution was refined by PHENIX.REFINE, the resulting molecular model manually adjusted into experimental electron density with COOT and further refined until reaching final *R*-work = 0.1812 and *R*-free = 0.2168 with 98.17% favored Ramachandran dihedrals. Reflection files and final models coordinates were deposited in the Protein Data Bank under accession codes 6I1C.pdb and 6I19.pdb, for CrTRXf2 and CrTRXh1, respectively. Protein models were analyzed with the webservers Structural Classification of Proteins (SCOPe), PDBeFold structure similarity, ConSurf server for the identification of functional regions in proteins, Pictorial database of tridimensional structures in the Protein Data Bank (PDBsum), and CATH Protein structure classification database. TRX surface electrostatic potentials were computed by the eF-surf algorithm [51] on Protein Data Bank Japan portal with the self-consistent boundary method, or by the Adaptive Poisson-Boltzmann Solver (APBS) Electrostatics plugin of PyMOL. Figures were drawn with PyMOL version 2.0.3 (The PyMOL Molecular Graphics System, Schrödinger, LLC).

### 2.4. Circular Dichroism (CD) Spectroscopy

CD analysis was performed at room temperature on a J-810 spectropolarimeter (Jasco, Tokyo, Japan). Samples of CrTRXf2 were prepared at a nominal concentration of 10 μM in 30 mM Tris-HCl buffer (pH 7.9). Reduced CrTRXf2 was obtained following 30 min incubation in the presence of a 10-fold molar excess of tris(2-carboxyethyl)phosphine (TCEP). The exact concentration of samples was determined from the absorbance at 280 nm (1 cm path-length) based on the theoretical molar absorption coefficients of 16,960 and 17,085 M^−1^ cm^−1^ for reduced and oxidized CrTRXf2, respectively [42]. The solutions were then transferred into a QS quartz cell with a 0.5 mm path length (Hellma, Milan, Italy) for far-ultraviolet (UV) CD measurements in the 250–195 nm spectral range, using a 20 nm min^−1^ scanning speed, a 4 nm response, a 2 nm spectral bandwidth, and an accumulation cycle of 3. Solvent-corrected CD spectra of reduced and oxidized CrTRXf2 were converted to molar units per residue (∆ε_res_) and analyzed using the BeStSel web server (http://bestsel.elte.hu [52,53]) to estimate the secondary structure contents.

## 3. Results

### 3.1. Sequence Analysis of Chlamydomonas TRX f2

To obtain insights on putative regions providing target specificity, *Chlamydomonas reinhardtii* TRX sequences were compared. Multiple sequence alignments revealed that Chlamydomonas TRXs exhibit low similarity ranging from ~21% to ~45% (Figure 1a). The highest homology was found between isozymes of chloroplastic f-type CrTRX (45.5%, f1 versus f2) and cytoplasmic h-type CrTRX (44.4%, h1 versus h2), whereas the lowest homology is observed between CrTRXh2 and CrTRXy (21.2%). The presence of fully conserved residues is restricted to the active site motif WCGPC containing the two catalytic Cys, and five amino acids (Figure 1a, highlighted in red). The latter strictly conserved residues were shown to be important for the function or the structure of diverse TRXs [54,55,56,57,58]. Comparison of CrTRXf2 sequence with plastidial and mitochondrial CrTRXs (CrTRX m, x, y, z, and o) revealed sequence identity in the ~22–29% range (Figure 1a), which is consistent with the low similarity shared by CrTRXs. By contrast, CrTRXf2 has a slightly higher homology when compared to h-type CrTRXs (35.2 and 30.5% with h1 and h2, respectively) (Figure 1a). When compared with other f-type TRXs from plants, the sequence identity increased to ~40%, AtTRXf1 having the highest homology (41.4%, Figure 1b). Moreover, 29 out of 125 amino acids in CrTRXf2 are fully conserved in all f-type TRXs (Figure 1b, highlighted in red), including the extra Cys located in position 65 of mature CrTRXf2. This homology analysis displayed a strong diversity, even for TRX from the same type. To gain further insights into the structural determinants of TRX specificity, we determined the crystal structure of two TRXs: CrTRXf2 and CrTRXh1. The latter is highly similar to previously described structures (RMSD = 0.287 Å to 1EP7.pdb [50]), and to structures described in a companion paper of this journal issue [59].

### 3.2. Chlamydomonas TRX f2 Folds as a Canonical TRX

**Figure 2:**
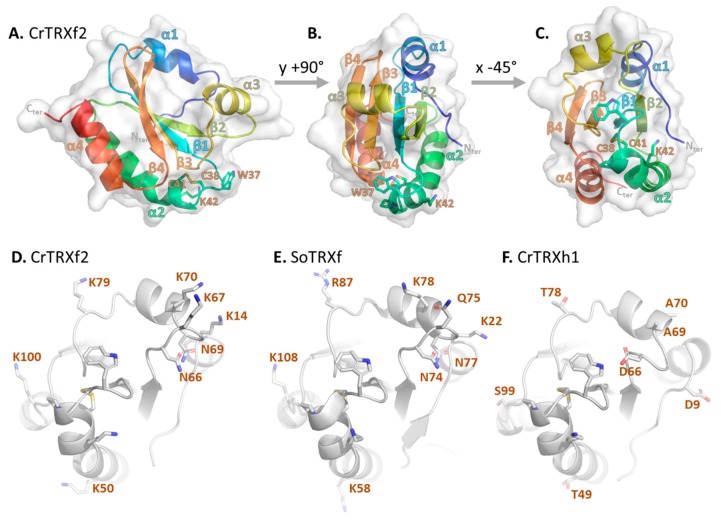
Crystal structure of *Chlamydomonas reinhardtii* thioredoxin f2 (CrTRXf2). (**A**–**C**) Three rotated projections of the crystal structure of CrTRXf2. The main chain is drawn as cartoon, colored from blue (amino-terminus) to red (carboxy-terminus). Side chains of the ^37^WCGPCK^42^ motif are drawn as sticks. The protein surface is displayed in transparent light gray. (**D**–**F**) Side chains of the ^37^WCGPCK^42^ motif and of electropositive residues conserved in (**D**) CrTRXf2 and (**E**) *Spinacia oleracea* SoTRXf, but not in (**F**) CrTRXh1, are shown as sticks on the cartoon-drawn main chain.

The crystal structure of CrTRXf2 was solved at 2.01 Å resolution. The closest structural match revealed by tridimensional comparison with the PDB archive is SoTRXf (PDB identifier: 2PVO) [60]. The structural alignment of the two enzymes gave an RMSD = 0.859 Å, despite CrTRXf2 exhibiting only 39.7% sequence identity with its spinach ortholog. As shown in Figure 2A–C, the secondary structures are organized from the N-terminus to the C-terminus, as follows (residue boundaries indicated in parentheses): α-helix 1 (16–23), β-strand 1 (29–34), α-helix 2 (39–54), β-strand 2 (59–64), α-helix 3 (70–76), β-strand 3 (83–88), β-strand 4 (91–97), α-helix 4 (101–111). β-strands order in the mixed β-sheet is 4–3–1–2, with β-strand 3 being antiparallel to the others. The β-sheet is sandwiched between α helices 1 and 3 on one side, and α-helices 2 and 4 on the other side. The overall structure is a flattened spheroid of 49 Å equatorial diameter and 25 Å polar diameter, and conforms to the classical TRX fold.

Structural alignments with CrTRXm (PDB ID: 1DBY) [61], and CrTRXh1 (this study) yield RMSDs of 0.927 Å and 0.953 Å, respectively. These close alignment scores further confirm the high structural similarity irrespective of a low sequence homology (24.2% and 35.2% identity with CrTRXm and CrTRXh1, respectively; Figure 1a). Hence, the cell requirements for TRX function maintained strong selection towards the TRX fold, despite the specialization of diverse types.

### 3.3. The Redox Site of CrTRXf2

In CrTRXf2, the first active site Cys (hereafter referred to as Cys_N_) is located at position 38 at the N-terminal kinked tip of α-helix 2. The second catalytic Cys (hereafter referred to as Cys_C_) is located at position 41, and it belongs to the same α-helix 2 (Figure 2A–C). In the CrTRXf2 structure, the catalytic Cys are covalently disulfide-bonded in accordance with the non-reducing conditions of the purification and crystallization procedures. Trp37, Gly39, Pro40, and Lys42 of the conserved ^37^WCGPCK^42^ motif arch over the disulfide bond, leaving two gates to interact with the solvent. In the solved crystal structure, the deep pocket on Tyr45 side of the bond is filled with water oxygens 20, 80, 89, 122, and 131 while the shallow crevice on the Pro82 side of the bond is occupied by water oxygen 110. The redox activity of the Cys pair either requires target disulfide docking on these gates, or rearrangement of the ^37^WCGPCK^42^ arch, to increase Cys_N_ thiol exposure.

Structural alignment of CrTRXf2 with CrTRXh1 revealed that the oxygen of water 20 of CrTRXf2 localizes 0.4 Å away from the corresponding oxygen of water 133 of CrTRXh1. In both structures, these equivalent water molecules hydrogen bond with Asp32/31 (CrTRXf2/CrTRXh1 numbering respectively) and Cys_N_, and this was previously characterized as a determinant for lowering the pK_a_ of Cys_C_ [50].

The third cysteine of CrTRXf2, located at position 65, is perfectly conserved amongst f-type TRXs (Figure 1b) and was shown to be modified by S-glutathionylation [62]. In our structure, Cys65 is likely in the thiol form, since its side chain points inward to a hydrophobic pocket formed by Phe33, Val63 and Ile78. Redox modification of Cys65 thus requires a local rearrangement of the TRX surface that is possible if loop 65–69 adopts an alternate conformation. Consistently, the equivalent loop on CrTRXh1 is flipped by 7 Å towards the domain core relative to the CrTRXf2 position, a conformation that is correlated with an additional flip of the N-terminal loop of CrTRXh1 by 4 Å in the same direction. These alternate conformations argue in favor of a flexibility of this region that may condition the redox regulation of Cys65 of CrTRXf2.

### 3.4. Comparison of the Secondary Structures of Oxidized and Reduced CrTRXf2

**Figure 3:**
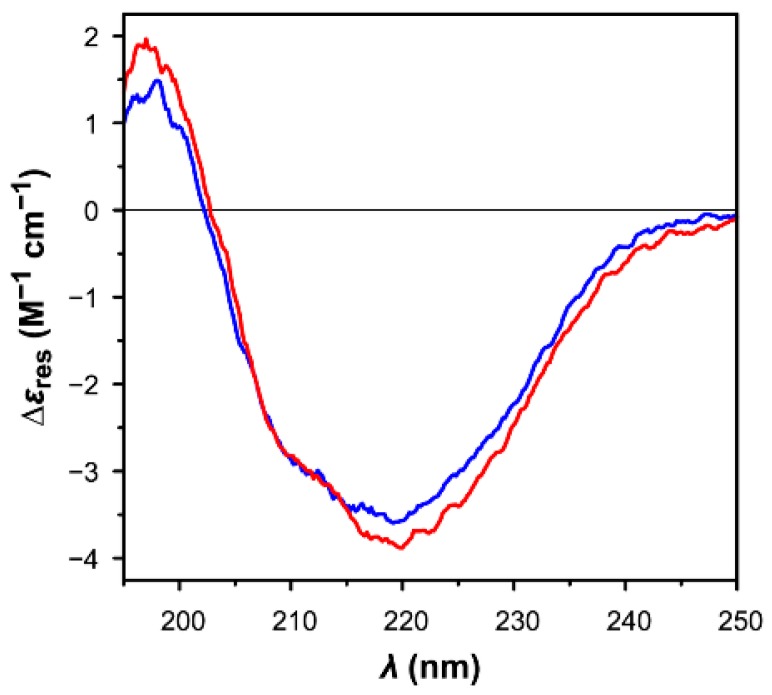
Far-ultraviolet circular dichroism spectra of reduced (blue line) and oxidized (red line) *Chlamydomonas reinhardtii* thioredoxin f2 (9.94 µM for reduced CrTRXf2, 9.28 µM for oxidized CrTRXf2) in Tris-HCl buffer (30 mM; pH 7.9).

The far-UV circular dichroism (CD) spectra of the reduced and oxidized forms of CrTRXf2 (Figure 3) differ slightly, with the former showing a more intense negative band at 220 nm, and an additional shoulder centered at ~210 nm. Nevertheless, the overall CD profiles of CrTRXf2 in both redox states are similar to those previously reported for other TRXs [63,64,65]. The secondary structure estimation given by the BeStSel algorithm predicts a lower percentage of α-helices (reduced: 18%; oxidized: 11%) and a slightly higher content in β-strands (reduced: 34%; oxidized: 29%), compared to the secondary structure of the crystal structure of oxidized CrTRXf2 (α-helix: 33%; β-strand; 19%) as calculated using the database of Define Secondary Structure of Proteins (DSSP) web server (https://swift.cmbi.umcn.nl/gv/dssp/ [66]), based on the full sequence of the His-tagged enzyme (125 residues). Even though some divergence can be expected between in-solution and solid-state protein structures [67], the observed variations probably have a different explanation, as detailed below.

On the experimental side, the absorption cut-off of the CD measurements did not allow to collect data below the 195 nm threshold, limiting the accuracy of the estimation. On the theoretical side, mixed α/β proteins still represent a tough challenge for the algorithms available for secondary structure estimations by CD spectroscopy, despite the recent and encouraging improvements provided by new methods. A textbook example of the huge discrepancies of results obtained by these approaches was indeed reported for TRXs [63]. The BeStSel fold recognition analysis, nevertheless, correctly predicts that both samples are structurally related to the class of mixed α/β proteins organized in a 3-layer (α/β/α) sandwich arrangement, in agreement with the typical tertiary structure of TRX (CATH classification 3.40.30.10; http://www.cathdb.info [68]).

### 3.5. Surface Specificities of CrTRXf2

CrTRXf2 cleft analysis revealed its 10 largest surface grooves, which include volumes of 785, 375, 295, 393, 262, 251, 206, 144, 127, and 138 Å^3^ for a total volume of 2976 Å^3^. An equivalent CrTRXh1 cleft analysis revealed its 10 largest surface grooves, which included volumes of 483, 462, 429, 452, 292, 300, 172, 81, 78, and 70 Å^3^ for a total volume of 2819 Å^3^. Hence, the surface topography of CrTRXf2 appears rougher than that of CrTRXh1, mainly because of its top single cleft of 785 Å^3^. Cavity sizes may control the hydrogen bonding of water molecules at the surface of the protein, thus modifying the local flexibility of TRX [69].

**Figure 4:**
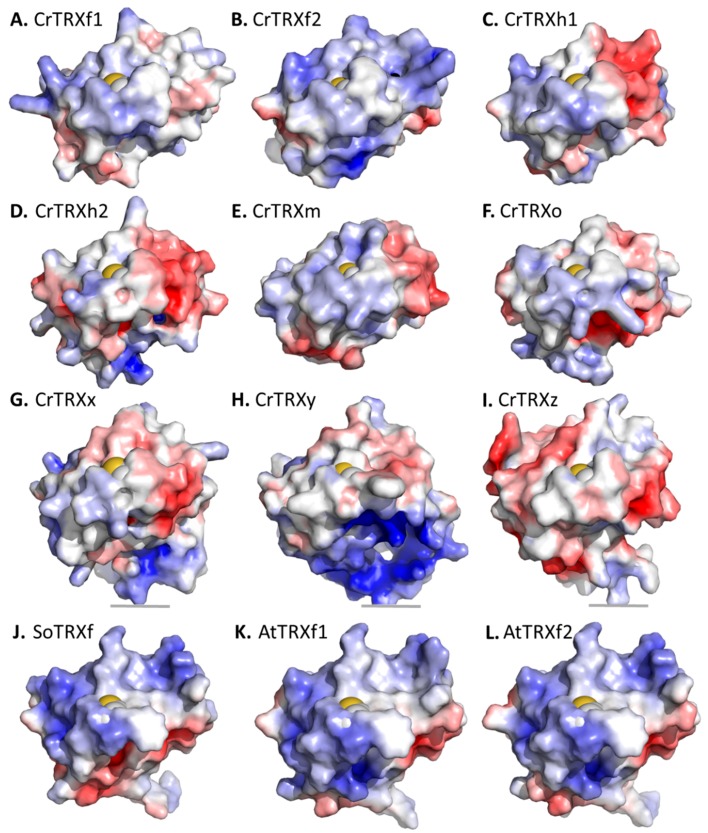
Electrostatic surface variation of plant thioredoxins. (**A**) PHYRE2 [47] homology model of chloroplast CrTRXf1. (**B**) Crystal structure of chloroplast CrTRXf2 (this study). (**C**) Crystal structure of cytosolic CrTRXh1 (this study). (**D**) PHYRE2 homology model of cytosolic CrTRXh2. (**E**) Nuclear magnetic resonance structure of chloroplast CrTRXm (Protein Data Bank identifier: 1DBY) [61]. (**F**) PHYRE2 homology model of mitochondrial CrTRXo. (**G**) PHYRE2 homology model of chloroplast CrTRXx. (**H**) PHYRE2 homology model of chloroplast CrTRXy. (**I**) PHYRE2 homology model of chloroplast CrTRXz. Electrostatic surface potentials were computed with the Protein Data Bank Japan webserver eF-surf [51] (red for electronegative, white for neutral, blue for electropositive). (**J**) Crystal structure of *Spinacia oleracea* TRXf (PDB ID: 1F9M). (**K**) PHYRE2 homology model of chloroplast *Arabidopsis thaliana* TRXf1. (**L**) PHYRE2 homology model of chloroplast *Arabidopsis thaliana* TRXf2. Electrostatic surface potentials were computed with the Adaptive Poisson-Boltzmann Solver (APBS) Electrostatics plugin in PyMOL software (red for electronegative, white for neutral, blue for electropositive). All structures were aligned in PyMOL. N-terminal active site Cys (Cys38 in CrTRXf2) side chain is displayed as spheres at the center of the projection (gold for thiol sulfur, white for carbon beta).

CrTRXf2 Cys38-Cys41 site is surrounded by basic side chains of six lysines (Lys14, Lys50, Lys67, Lys70, Lys79, Lys100) and two hydrogen bond donor side chains (Asn66, Asn69) (Figure 2D). These positions align with positively charged (Lys22, Lys58, Lys78, Arg87, Lys108) or hydrogen bond donor side chains (Asn74, Asn77) of the spinach TRX f ortholog (PDB ID: 1FAA, 1F9M) [60] (Figure 2E). CrTRXf2 electropositive patches locate on the α-helix 3 side of the disulfide, and on the β-strand 2 side of the disulfide. These eight positions form an electropositive crown around the active site, conserved in both plant and algal enzymes (Figure 2D,E). In striking opposition, the corresponding residues in CrTRXh1 are negatively charged (Asp9, Asp66), electronegative (Thr49, Thr78, Ser99), or neutral (Ala69, Ala70) (Figure 2F). CrTRXh1 corresponding surface displays an electronegative potential on the α-helix 3 side of the disulfide and of negligible polarity on the β-strand 2 side of the disulfide. Hence, despite the strong conservation bias for a common fold and active site composition, CrTRXf2 and CrTRXh1 display distinct electrostatic potential on their solvent accessible surface (Figure 4B,C).

The electropositive surface of CrTRXf2 compares with the equivalent regions of modelled CrTRXf1, although the latter possess a more neutral character (Figure 4A,B). If experimentally confirmed, this may explain a more stringent specificity of CrTRXf2 than CrTRXf1 for equivalent targets. This f-type electropositive character is maintained in land plant enzymes (Figure 4J–L). CrTRXh1 and the modelled CrTRXh2 both present mixed polarities, confirming the significant difference of these cytoplasmic isoforms compared to the f-type CrTRXs (Figure 4C,D). The surface of chloroplast CrTRXm is closer to the h- than f-type CrTRXs (Figure 4E), while modelled CrTRXx, CrTRXy, and CrTRXz all present neutral or electronegative surfaces around the catalytic cysteines (Figure 4G–I). The modelled mitochondrial CrTRXo appears similar to CrTRXh1 and m (Figure 4F).

### 3.6. Surface Specificities of TRXf Targets

**Figure 5:**
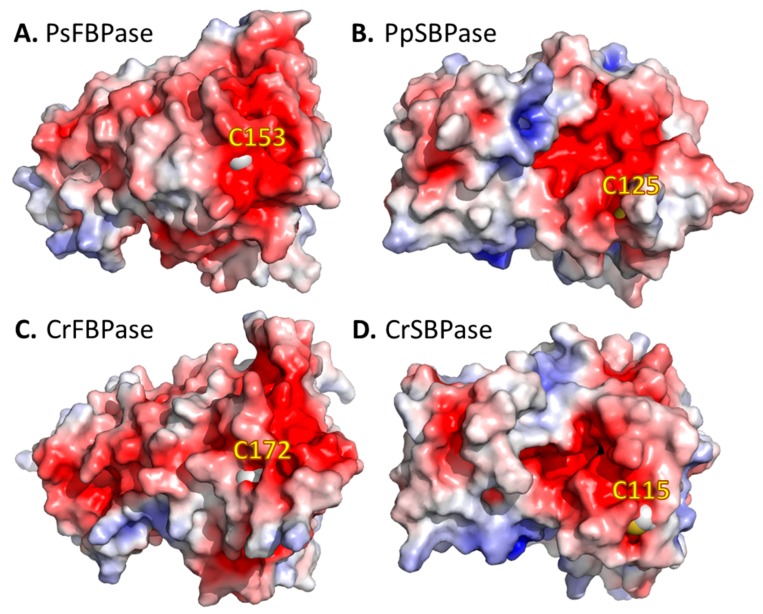
Electrostatic surface potential of thioredoxin f targets. (**A**) Crystal structure of *Pisum sativum* FBPase (PsFBPase, PDB ID: 1D9Q, [70]). (**B**) Crystal structure of *Physcomitrella patens* SBPase (PpSBPase, PDB ID: 5IZ1, [32]). (**C**) PHYRE2 homology model of *Chlamydomonas reinhardtii* FBPase (CrFBPase). (**D**) PHYRE2 homology model of *Chlamydomonas reinhardtii* SBPase (CrSBPase). Target cysteines are displayed as spheres (white for carbon beta, gold for thiol sulfur). Electrostatic surface potentials were computed with the APBS Electrostatics plugin in PyMOL (red for electronegative, white for neutral, blue for electropositive).

Protein–protein interaction with specific CrTRXf2 targets would involve a complementary electronegative surface. Indeed, we observed an extended continuous electronegative surface around the cysteines of the two f-type TRXs targets, FBPase and SBPase (Figure 5). Moreover, the electronegative character of targets was identically observed in orthologues from both vascular (*Pisum sativum* [70], Figure 5A) and non-vascular land plants (*Physcomitrella patens* [32]), accounting for the conservation of this structural feature over speciation and evolution. We applied molecular docking simulations to orient possible interactions of CrTRXf2 with pea FBPase [70]. The FRODOCK algorithm [71] suggested 10 docking models of CrTRXf2 on the pea FBPase surface, four placed Cys_N_ of CrTRXf2 in the vicinity of target Cys153. These plausible solutions all bring the electropositive surface of α-helix 3 in contact with the target electronegative surface. Contrarily, the alignment of CrTRXh1 at the docked positions unfavorably joins the negative patches of both surfaces.

Upon the recognition of electro-complementary surfaces, the actual reduction of target disulfide requires a rearrangement of TRX to bring Cys38 in bonding distance to target Cys153. Alignment of CrTRXf2 structure with *Hordeum vulgare* TRXh2 complexed to the model target barley α-amylase/subtilisin inhibitor (BASI) [58,72] suggests that loop 35–38, loop 65–69, and α-helix 1 undergo most of the conformational variation, while the rest of the protein remains unaffected. Molecular structures determined by X-ray crystallography attribute an isotropic displacement B-factor to each atom of the refined model, the value of which quantifies the thermal vibration during data collection, and the variation of the atom position in the unit cell. The β-sheet core of both CrTRXf2 and CrTRXh1 display the lowest B-factor values, in accordance with a stably (thermostable) folded globular domain [73,74]. Both the N-terminal and C-terminal residues of the two TRXs display high B-factors, accounting for the poor resolution at the extremity of the modelled electron density. In the solved CrTRXf2 crystal structure, the complete α-helix 2, the first turn of α-helix 4, and the loop downstream of α-helix 1 are formed by atoms of the highest B-factor in the model. Meanwhile, the solved crystal structure of CrTRXh1 displays the highest B-factors on the complete α-helix 2 and the first turn of α-helix 4, but not at the loop downstream of α-helix 1. The pentapeptide ^23^QQQDT^27^ of CrTRXf2 appears as a specific site of local flexibility. This hinge at the basis of α-helix 1 would appropriately support its movements upon target recognition.

## 4. Discussion and Conclusions

The resolution of the crystal structure of CrTRXf2 confirms the highly conserved structure of the TRX fold, despite it having a low sequence identity (Figure 1 and Figure 2). The newly characterized Chlamydomonas TRX f2 has the same secondary structure composition and wiring diagram as CrTRXh1 when used as a reference. In addition, CD spectra revealed minor conformational changes in CrTRXf2, when analyzed under both oxidized and reduced forms. The active site is centered on the pair of Cys38 and 41 near the solvent-exposed surface of the protein. In the CrTRXf2 structure, these cysteines are disulfide bonded. The Cys pair points inward to a peptidic arch composed of the conserved ^37^WCPGCK^42^ motif. The motif contributes to restrict the accessibility of the disulfide for its reduction by ferredoxin–thioredoxin reductase and its oxidation by TRX targets.

Despite the high functional and structural similarity in the TRX family, chloroplastic f-type TRXs specifically or preferentially activate Calvin–Benson cycle enzymes (i.e., FBPase, GAPDH, SBPase, PRK, and PGK). The structures of these target enzymes have been solved, but not in complex with TRX f, which limits our understanding of the molecular interactions and contact sites between the two molecules. Nevertheless, the solved structure of a complex between barley TRX h2 and the α-amylase/subtilisin inhibitor (BASI) stabilized through a mixed-disulfide bond corresponding to a reaction intermediate serves as a working model [72]. The structure of CrTRXf2 aligns with barley TRXh2 complexed to BASI (RMSD = 1.303 Å). Cys_N_ is situated slightly away from its internal orientation compared to free TRX, towards an outward exposure that allows for its interaction with target Cys148. The target protein attacks the Tyr45 side of TRX in the deeper pocket of the ^37^WCGPCK^42^ arch. In the course of complex formation, water molecules 104, 105, 106, 110, and 160 of CrTRXf2 model will leave the surface, to allow for target accommodation. Such an entropic effect of enzyme–substrate complex formation may be tested by in vitro experiments such as isothermal titration calorimetry [75].

Detailed comparative analysis of the crystal structures of CrTRXf2 with other TRXs revealed that the f-type specifically (i) forms a rougher surface with larger cavities, (ii) orients a crown of electropositive patches on the two opposite sides of the active site disulfide, and (iii) adopts a local flexible hinge downstream of α helix 1. These specific structural features of CrTRXf2 should guide the recognition of specific targets by facilitating target space accommodation, flexibility, and electrostatic interactions. These results are consistent with previous studies that suggested a major role for electrostatic interactions in the TRX–target interactions in chloroplasts [29,76]. Modelled CrTRXf1 displays a surface of lower electropositive potential than CrTRXf2, suggesting that CrTRXf1 is less efficient at targeting Calvin–Benson enzymes for reduction. This hypothesis should be tested by comparing CrTRXf1 and CrTRXf2 activities towards Calvin–Benson enzymes, and by the determination of CrTRXf1 experimental structure. To gain further insights into the structural determinants of TRX specificity, future studies should be aimed at solving the structure of TRX-target complexes and engineering the different TRX types, notably by altering the distribution of charges around the active site. Such knowledge may allow predicting the TRX dependence of the numerous putative targets identified by proteomics [23], and possibly rationalize the design of TRXs with predictable specificities. To test these hypotheses in vivo in *Chlamydomonas reinhardtii*, new tools are available [77,78] that should accelerate prototyping of artificial TRX.

## Figures and Tables

**Table 1:** Crystallographic data collection and refinement statistics.

	CrTRXf2	CrTRXh1
Wavelength (Å)	0.9762	0.9677
Resolution range	42.85–2.01 (2.082–2.01)	36.42–1.378 (1.427–1.378)
Space group	I 2 2 2	P 31 2 1
Unit cell	65.383 97.475 139.545 90 90 90	48.76 48.76 143.97 90 90 120
Total reflections	57,413 (5796)	83,812 (8115)
Unique reflections	29,702 (2973)	41,955 (4085)
Multiplicity	1.9 (2.0)	2.0 (2.0)
Completeness (%)	98.74 (99.80)	99.80 (98.81)
Mean I/sigma (I)	6.69 (0.95)	25.45 (3.98)
Wilson B-factor	34.89	16.29
R-merge	0.0697 (0.7309)	0.01107 (0.1238)
R-meas	0.09857 (1.034)	0.01565 (0.1751)
R-pim	0.0697 (0.7309)	0.01107 (0.1238)
CC1/2	0.997 (0.485)	1 (0.968)
CC *	0.999 (0.808)	1 (0.992)
Reflections used in refinement	29,749 (2970)	41,938 (4085)
Reflections used for R-free	1998 (200)	2095 (204)
R-work	0.2262 (0.3131)	0.1812 (0.2281)
R-free	0.2638 (0.3606)	0.2168 (0.2690)
CC (work)	0.951 (0.709)	0.964 (0.914)
CC (free)	0.915 (0.649)	0.946 (0.940)
Number of non-hydrogen atoms	2764	2033
Macromolecules	2585	1638
Solvent	179	395
Protein residues	318	222
RMS (bonds)	0.008	0.005
RMS (angles)	0.97	0.77
Ramachandran favored (%)	98.34	98.17
Ramachandran allowed (%)	1.33	1.83
Ramachandran outliers (%)	0.33	0.00
Rotamer outliers (%)	0.34	0.60
Clashscore	5.18	4.24
Average B-factor	39.23	19.69
Macromolecules	39.04	17.65
Solvent	42.01	28.15
